# Combinational therapeutics to combat cancer

**DOI:** 10.6026/97320630017673

**Published:** 2021-07-31

**Authors:** Ahmed Al Otaibi, Subuhi Sherwani, Eida Mohammed Alshammari, Salma Ahmed Al-Zahrani, Wahid Ali Khan, Abdulmohsen Khalaf Dhahi Alsukaibi, Sourabh Dwivedi, Shahper Nazeer Khan, Mohd Wajid Ali Khan

**Affiliations:** 1 Department of Chemistry, College of Sciences, University of Hail, Hail-2440, Saudi Arabia; 2 Department of Biology, College of Sciences, University of Hail, Hail-2440, Saudi Arabia; 3 Department of Clinical Biochemistry,College of Medicine, King Khalid University, Abha-62529, Saudi Arabia; 4 Department of Applied Physics, Aligarh Muslim University, Aligarh-202002, U.P., India; 5 Interdisciplinary Nanotechnology Centre, Aligarh Muslim University, Aligarh-202002, U.P, India; 6 Molecular Diagnostic and Personalised Therapeutics Unit, University of Hail, Hail-2440, Saudi Arabia

**Keywords:** biologically active molecules, T cells, cancer, combinational therapeutics, synergistic

## Abstract

Mono-therapeutics is rarely effective as a treatment option, which limits the survival of patients in advanced grade aggressive cancers. Combinational therapeutics (multiple drugs for multiple targets) to combat cancer is gaining momentum in recent years. Hence, it is of interest to document known data for combinational therapeutics in cancer treatment. An amalgamation of therapeutic agents enhances the efficacy and potency of the therapy. Combinational therapy can potentially target multiple pathways that are necessary for the cancer cells to proliferate, and/or target molecules, which may help cancer to become more aggressive and metastasize. In this review, we discuss combinational therapeutics, which include human γδ T cells in combinations with biologically active anti-cancer molecules, which synergistically may produce promising combinational therapeutics.

## Background:

Cancer is a disease characterized by abnormal and unchecked growth of cells, causing morbidity and mortality globally, with approximately 19.29 million new cases in 2020 according to World Health Organization (WHO) [1]. A 70% increase in new cases is expected over the next two decades. Cancer was estimated to be the second leading cause of deaths worldwide in 2020, and was responsible for 9958133 deaths [1]. Estimates for 2020, indicate approximately 1.8 million newly diagnosed cancer cases and 606,520 cancer related deaths in the US [2]. According to the WHO, Saudi Arabia reported 10.2% deaths due to cancer in 2012. A substantial number of cancer related deaths in adults were due to colorectal (males; 12.5%) and breast (females; 18.7%) cancers [3]. As per statistics for 2014, the most common types of cancers among Saudi children of both sexes were leukemia (34.6%) and cancers of the brain and nervous system (15.1%) [4]. A single therapeutic strategy may not be as effective. Therefore, there is a need for combinational therapies that act simultaneously, targeting different pathways to inhibit and/or kill tumor cells [5]. Bioengineers in collaboration with medicinal chemists have successfully discovered effective and safe clinical candidates capable of acting on innovative targets [6]. Medicinal chemistry and adoptive cell therapy are two of the most advanced areas of research developing a number of potential therapeutics for cancer treatments. For the systemic treatments of disseminated cancers, new biomolecules are needed as effective therapeutic agents to combat cancer. Scientists are developing complex active organic molecules with specific structural activities to target specific biological functions. An example of one such molecule is Imatinib, which represents a landmark innovation in cancer treatment [6]. Scientists are focusing on studying the tumor genetics or epigenetic dysregulation of cellular processes, as abnormalities in these processes might be responsible for cancer origin, and consequently lead to the discovery of new molecular targets for treatment as well as development of biologically active molecules [6,7]. Cell based cancer therapeutics can be carried out using in *in vitro* expanded immune effector cells, with transference of these activated immune cells to patients [8]. Elimination of tumor cells can be achieved by targeting the tumor cells or stimulation of the immune response [9]. Human γδ T cells vigorously contribute to the anti-tumor immune response against several tumors (lymphoma, myeloma, melanoma, colorectal, colon, breast, ovary and prostate cancers) [10]. Biologically active compounds and γδ T cells are ideal candidates for use in cancer therapeutics [8,11-13]. Both candidates are easily producible, stable, and can be generated in large numbers or amounts. Also, previous data supports that both can be applied potentially to all neoplastic diseases [6,7,10].

## Biologically active molecules:

Medicinal chemistry is unique in generating molecules with specific structural activity relationships to target specific biological functions [14]. The diversity of the available biological targets necessitates researchers to focus their efforts on specific target areas. Biologically active molecules can target immune suppressive and activation pathways in both innate and adaptive immune cells. These molecules can be taken orally and can cross the cell membrane effectively to target intracellular molecules [14,15]. There are many different types of immune cells, receptors and molecular pathways implicated in diverse types of tumors, offering numerous potential targets for these small molecules [16]. Another important advantage of small molecules is their low cost, which could enable access to a greater spectrum of patients. Imatinib represents a landmark in cancer treatment. It is among the most successful biological small molecules for anti-cancer treatment receiving wide acknowledgement from the health care community due to its specific targeting ability [17]. Imatinib inhibits proliferation in BCR-ABL positive cells and promotes apoptosis in these cells [17]. The use of Imatinib in chronic myeloid leukaemia CML patients as a targeted therapy has markedly lowered the death rate [18]. The last few years have witnessed scientists working across a variety of biological target areas to develop medicinal chemistry methodologies to rapidly access biologically active compounds [19,20].

## Potential biologically active anticancer molecules:

Several research studies focus on new organic synthetic methods for developing high yielding organic compounds and drugs under environmentally benign conditions with advantages over traditional methods [21]. New methods using environmentally friendly procedures are also characterized by reduced synthesis cost and decrease in waste by-products [21]. Modern techniques used by researchers for synthesizing bioactive compounds include microwave-assisted synthesis, solid phase supported solvent-free synthesis, reaction with organocatalyst, one-pot multicomponent reactions and sonochemical synthesis [22]. Pharmaceutical companies are also working on to improving chemicals used in drug development to minimize environmental hazards. Some of the important anti-cancer molecules synthesized and their target cancers are given in Table 1(see PDF) [22].

The global oncology trend report (2018) revealed that the global spending on cancer medication and therapeutic and supportive care rose from $96 billion in 2013 to $133 billion globally in 2017 [38]. Overall, the global oncology therapeutic medication market is estimated to reach $200 billion by 2022. An average of 12-15% growth is expected within the U.S. market over the next five years, reaching approximately $100 billion by 2022 38]. Therefore, new chemotherapeutic agents with lower toxicity, superior efficacy and better selectivity are required with a systematic approach. Furthermore, in the review we discuss the production and efficacy of organic molecules with anticancer potential in cancer cell lines.

## Synthesis and anti-cancer activity of biologically active molecules

Utilizing current medicinal chemistry approaches is possible in the synthesis of various complex molecules in comparatively short periods of time and with efficient use of energy [21]. There has been a drastic growth in the use of microwave for small molecules synthesis in the last 10 years [20]. Microwaves have wide applications in chemical transformations and have been adopted in organic molecule synthesis [22]. Microwave acceleration can be applied in chemical transformation that depend on heating, and consequently boosts the rates of reaction, yields, and decreases the time taken by a reaction. Gedye [39] and Giguere [40] were first to report the use of microwave irradiation as an alternative heating method in synthetic chemistry in 1986. There is a direct association between efficiency of microwave technology and the capacity of the reaction mixture for absorbing the microwave energy [41]. This technology has found its application within technologically advanced areas, including high-throughput parallel synthesis and combinatorial medicinal chemistry [42]. The use of microwave irradiation in a laboratory setting offers advantages such as selective material heating, efficient heating, increased rate of reaction, enhanced conversion of starting materials to product and thus a reduction of waste generated relative to traditional heating of small scale batch reactions [22,43,44]. The ability to safely superheat reaction mixtures, thus accelerating the reaction, is a significant advantage of microwave irradiation. In a recently published method by Otaibi et al. [22], synthesis of some important small molecules using microwave reactions and their cytotoxicities on testing are given in Table 2(see PDF).

## Cancer immunotherapy:

Immune cells play an integral part in tumor cell control (immunosurveillance), and immune defects are frequently associated with cancer development and disease progression [8]. Consequently, corrective measures aimed at restoring anti-tumor immunity are a major focus in current research to develop novel cancer immunotherapies [8]. T cells as the main effector cells characterize the immune response against tumors. The innate immune system activates T cells through positive and negative costimulatory molecules. Alternative strategies involve chimeric antigen receptors (CAR)-expressing immune effector cells as well as immune checkpoint inhibitors (Imatinib, programmed death 1 (PD-1), monoclonal antibodies against cytotoxic T lymphocyte associated protein 4 (anti-CTLA-4 Abs) and B7 ligands). These are recognized as promising new tools in the arsenal of immune-based cancer therapeutics [6,17, 18,45]. PD-1 and programmed death 1 ligand (PDL-1) axis blocks TCR and CD28 signaling and inhibit the optimal functioning and antitumor activity of tumor specific T cells [46, 47]. Small molecules, which can work as antagonists for PD-1/PD-L1, may be useful in enhancing the activity of cytotoxic T lymphocytes. A biologically active small molecules CA-170, which is an antagonist for PD-L1, PD-L2 and V-domain Ig suppressor of T cell activation, has been recently evaluated in a Phase I trials. CA-170 was orally given to advanced solid tumors or lymphoma patients who showed some progress in response to treatment or were non-responsive to other available therapies. Administration of CA-170 induced activation of effector T cell proliferation and secretion of cytokines [46]. In another clinical trial, small molecules cisplatin/carboplatin with humanized antibody pembrolizumab (MK-3475), was given to patients with advanced or metastatic nonsqamous non-small cell lung cancer and showed prolonged survival of patients as compared to the patients given cisplatin/carboplatin chemotherapy alone [48]. Small molecules targeting the PD-1/PD-L1 pathway are a less effective treatment option as compared to mAbs. Further work in design and development of small molecules needs to address problems of the hydrophobic PD-1/PD-L1 interface. Another clinical trial conducted in December 2017, was based on a biologically active molecule imiquimod in combination with a humanized antibody pembrolizumab, which is used in cancer immunotherapies. Patients with unresectable cutaneous melanoma received pembrolizumab intravenously on day 1 and imiquimod was applied cutaneously on day 1-5. This was repeated every 21 days and approximately 35 cycles of the course was given in conditions where no further progression of disease or unacceptable toxicity were observed. For the next two years the patients were under observation and the trial is set to end in February 2023 [46]. Recent discoveries suggest that combination of immunotherapies will likely be required to enhance and broaden the anti-tumor activity of immune checkpoint inhibition [49]. However, these immune checkpoint inhibitors are effective in rapidly growing tumors. Antigen presentation function of human blood derived γδ T cells (γδ T-APC) has been established [50], which can potentially work as cellular vaccines, to overcome many of the problems associated with moDCs. In fact, γδ T-APCs are (functionally) robust, affordable, feasible for routine use to most types of tumors (irrespective of HLA haplotypes) [11].

## γδ T cell immunotherapy:

γδ T cells are unique unconventional T cells, which are distinguished from the major T cell subset i.e., αβ T cells, by the T cell antigen receptor (Vγ9Vδ2-TCR), which they express on their cell surface [11, 12]. γδ T cells fulfil numerous important functions in immunity, including cytokine production in response to microbial challenges, mobilization of other types of immune cells and tumor cells killing (in vitro) [11,51]. An ex vivo expansion strategy of γδ T cells has already been established, which is an important tool for cancer immune-therapeutics. Firstly, peripheral blood mononuclear cells (PBMCs) were isolated from peripheral blood and stimulated ex vivo using zoledronic acid [8,12]. These features of γδ T cells led to numerous clinical studies using γδ T cells as effector cells in the treatment of cancer patients as given in Table 3(see PDF), including leukemia [52], colorectal carcinoma [53][54], renal cell carcinoma, melanoma, and acute myeloid leukemia [54].

The above finding revealed that large numbers of γδ T cells were well tolerated although clinical benefits have not been fully ascertained [63]. γδ T cells recognize these phosphoantigens in an HLA-unrestricted fashion; hence the activation does not depend on recognized antigen presenting cells (APCs) e.g. DCs. Some tumor cells yield high IPP concentration of a metabolite of mevalonate pathway, which can be recognized by γδ T cells [64]. The administration of the nitrogen containing bisphosphonates drugs (such as pamidronate and zoledronate), which are prescribed for patients of osteoporosis and hypercalcemia of malignancies, leads to enhanced intracellular levels of IPP due to inhibition of an enzyme of the mevalonate pathway i.e. farnesyl diphosphate synthase. Increase in IPP concentration can subsequently lead to activation and expansion of human γδ T cells [65,66]. Wada et al. [61] used ex vivo expanded γδ T-cell for treatment of malignant ascites caused by peritoneal dissemination of gastric cancer. An intraperitoneal injection of γδ T-cell allowed access to the peritoneal tumor cells. Computed tomography revealed significant reduction of ascite volume in two out of seven patients. The most commonly observed treatment related side effects were mild fever and zoledronate-induced hypocalcemia [61]. Therefore, infusion of γδ T cells clearly recognize tumor cells and show cytotoxicity against them *in vivo* on gaining access to tumor cells.

## Potential biological active molecules and γδ T cell combinational anti-cancer models:

**Figure 1:**
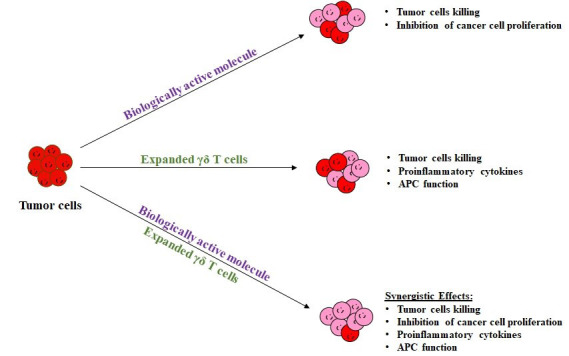
Synergistic approach of combinational therapeutic effect as compared with single therapeutic treatments

Cancer cell progression is not easy to understand and possess complex multiple pathways, which include numerous interconnected molecules. Single drug or vaccine has limitations in countering the complexity of tumor pathogenesis [5]. Combinational therapeutics is a new approach and provides many effective treatments for various cancers [67]. However, it is important to check the toxicities of combinational therapeutics before administration. Preclinical studies are crucial, and should be conducted sincerely before clinical trials. There are promising indications related to circumstances with protuberant participation of inhibitory immune cells (Treg cells, myeloid-derived suppressor cells), success with small biological molecules, which are immune checkpoint inhibitors [anti-CTLA-4 Abs (Ipilimumab), anti-PD-1 Abs (Nivolumab)], which may prove promising [64]. Combination therapy with vaccines of γδ T cell and immune checkpoint inhibitors may produce synergistic outcomes, e.g. inhibitory immune cells blockade by immune checkpoint inhibitors may assist the stimulatory effect of γδ T cells [64,68]. This improves effector responses of tumor-specific T-cells as well as long-lived immunosurveillance T-cell development. γδ T cell immunotherapies will not be restricted to a particular type of cancer as most human cancers arouse T-cell responses. New drugs for combination therapeutics evolved using bioengineering strategies and one of the most common products are trastuzumab, a monoclonal antibody. It is utilized in combination with cisplatin to inhibit the progression of gastric cancer [69]. Trastuzumab enhances cellular apoptosis by suppressing the DNA repair pathway and the PI3K-AKT pathway [70,71]. Cisplatin induces DNA damage and apoptosis, which may be attenuated by DNA repair systems [72]. Plant based molecules have been widely used from ancient time and would be important candidates for combination therapeutics. Paclitaxel, a compound obtained from the Taxus brevifolia, is widely used in combination with ramucirumab (human monoclonal antibody). It has been found to expressively increase the survival of patients with gastric cancer around the globe [73]. The combined drugs Paclitaxel and bortezomib also show better survival of patients with non-small cell lung cancer [74]. Extract of Viscum album L. has extensively been utilized for integrative oncological approaches [75,76]. The drug Abnoba Viscum enhances γδ T cells, which trigger the release of cytotoxic granules and promote production of IFNγ and TNFα. The utilization of plant-based products might be promising resources for novel combination therapeutics development. Additionally, the administration of dosages in combination therapeutics is an important aspect utilizing new technologies for delivery, such as the CombiPlexR platform, which ensures the efficient delivery of combination treatments. The CombiPlex platform was successfully used for the liposome based drug delivery of cytarabine and daunorubicin. The toxicity and increase of multidrug resistance in normal healthy cells is a major constraint in combination chemotherapy [77]. Hence, combinational therapeutics has the potential to overcome molecular heterogeneity in patients diagnosed with various cancers. As depicted in Figure 1, the effect of combinational therapeutics (γδ T cells and biologically active anti-cancer small molecules) is better as compared to monotherapeutics. We expect that combinational therapeutics can work synergistically and may exhibit promising anti-cancerous effects. Recently, we tested combinational effect of biologically active molecules (α-amino amide derivatives) in combination with in vitro expanded γδ T cells [13].

## Conclusion:

The use of potential methodologies to develop small biological active molecules with standardized reactions has been discussed in this review. We document known data for combinational therapeutics in combating cancer. These molecules exhibit potential anti-tumor activities and remain stable at room temperature. Biologically active molecules have inherent advantages over adaptive immunotherapies, as these molecules can reach a wide spectrum of molecular targets, including intracellular targets or those present deep in the tumor milieu. Human γδ T cells exhibit expression for cell surface markers for antigen presentation, co-stimulation, cell adhesion, cell activation and effector state, all together with tumor killing activity. Thus, the combination of both small molecules and γδ T cell are an important consideration for the future development of cost-effective combinational therapeutics. Interesting outcomes would be expected due to the concomitant synergistic effects of biological active small molecules and γδ T cell, driving efficient tumor cytotoxicity. In future small molecules can be engineered to perform dual functions; first as we mentioned in this review to inhibit cancer cell proliferation and second to activate *in vivo* γδ T cells.
